# Noninvasive identification of molecular biomarkers of hepatocellular carcinoma in HCV-Egyptian patients

**DOI:** 10.1186/s43046-023-00170-7

**Published:** 2023-05-01

**Authors:** Ahmed Daif, Mahmood A. Al-Azzawi, Moustafa A. Sakr, Hisham A. Ismail, Mahmoud Gadallah

**Affiliations:** 1 Molecular Diagnostics and Therapeutics Department, Genetic Engineering and Biotechnology Research Institute (GEBRI), University of Sadat City, Sadat City, Egypt; 2 Department of Forensic Science, College of Science, AlKarkh University of Science, Baghdad, Iraq

**Keywords:** HCC, Biomarkers, AFP, Egyptian patients, ROC curve

## Abstract

**Background:**

This study was performed to investigate the expression of different biomarkers in patients with hepatocellular carcinoma and its connection with detective biomarkers. To achieve this objective, seventy subjects were examined in this study, sub-grouped to forty HCC patients and thirty HCV-affected patients with matched thirty healthy individuals. The study involved several groups of participants who were matched based on their age and gender.

**Methods:**

The expression pattern of biomarkers was monitored by quantitative polymerase chain reaction (qRT-PCR). Finally, we utilized a ROC curve to investigate the predictive accurateness of those distinct biomarkers as well as a traditional tumor marker, AFP, in detecting HCC cases.

**Results:**

The baseline biomarker expression levels were markedly greater in HCC patients than in those affected by HCV or healthy subjects. We stated that the sensitivity and the specificity of the different biomarkers alone did not improve than that of AFP alone. When comparing AFP with different biomarkers, the diagnostic validity improves only when combining with CK-1.

**Conclusions:**

Overall, our results indicate that CK-1 mRNA expression could help as a noninvasive tumor biomarker for HCC prognosis and diagnosis when combining with AFP.

## Background

Hepatocellular carcinoma is the sixth most widespread malignancies in the globe and the third most lethal [1], which accounts for 70–85% of cases [2]. HCV, HBV, alcoholic and nonalcoholic liver disease, and fatty liver disease consider the major etiological HCC risk factors. These dangerous factors contribute to the development and cirrhosis progression, which is manifested in approximately 80–90% of HCC-affected patients. Outstanding to the high infection incidence of HCV and HBV in Egypt, the HCC occurrence rate has increased in the last 10 years [3]. The situation in Egypt is severe with the world’s highest prevalence due to an epidemic outbreak of HCV. The 5-year aggregate risk of HCC development in people with cirrhosis varies between 5 and 30%, depending on the cause (high in HCV), ethnicity or area (more higher in Asians), and degree of cirrhosis (high in decompensated illness) [4]. Although CT and MRI are more effective at detecting HCC than ultrasonography, they are correlated with a higher proportion of false positives [5]. Although modern treatments such as liver transplantation, surgical resection, and ablation therapy are now accessible [6], and despite the improvement of molecular-targeted medicines like sorafenib, patients with advanced HCC have a low 5-year overall survival rate [7]. The high frequency of metastasis and recurrence following local handling is the fundamental explanation for advanced HCC’s poor survival rate [8]. Because it is low cost and straightforward to carry out, and easily obtainable, AFP quantification is widely used for surveillance of HCC. AFP level alone (without liver ultrasonography) is not advisable as an HCC monitoring test due to its poor specificity and sensitivity for HCC detected. AFP has a low sensitivity for diagnosing HCC at 20 ng/ml a serum cut-off level, ranging from 25 to 65% [9]. The lack of a distinct biomarker for HCC with greater specificity and sensitivity than AFP highlights the need for further biomarkers responsible for human hepatocarcinogenesis to be developed.

Biomarkers provide a wide range of potential applications in cancer, including differential diagnosis, screening, prognosis, and disease progression and monitoring [10]. Diagnostic biomarkers can be used to optimize disease diagnosis. Different feature selection strategies are often used to tackle diagnostic biomarker discovery, which is the process of identifying key traits that can differentiate tumors from healthy samples [11]. Many researches have focused on finding investigative biomarkers by looking for differentially expressed genes (DEGs), which are the most useful genes from a huge number of inappropriate ones. Yin et al., for example, identified biomarkers for hepatocellular carcinoma by combining DEG screening with the weighted genomic co-expression network analysis technique.

Contemporary gene profiling analysis enables the identification of particular genetic patterns as well as the molecular mechanisms implicated in HCC [12, 13]. This method may aid in the identification of good screening indicators for HCC. Multiple reports that used this technique outlined numerous genes and their proteins that were considerably overexpressed in tumor tissues and showed promise as novel HCC biomarkers [14, 15].

New insights for HCC diagnosis may come from multi-omics, which includes transcriptomics, genomics, epigenomics, glycoproteomics/glycomics, proteomics, and metabolomics. More data has emerged that circulating tumor DNAs (ctDNAs) and their epigenetic modifications can be employed as credible biomarkers in the fields of genomics and epigenomics [16–18]. In terms of transcriptomics, noncoding RNAs and mRNAs showed major alterations (lncRNAs, circRNAs, miRNAs) [19]. Potential protein biomarkers for the identification of HCC have been found in proteomics, including Golgi protein-73 (GP73), heat shock protein 90 (Hsp90), alpha-1-fucosidase (AFU), Dikkopf-1 (DKK1), midkine (MDK), osteopontin, and des-gamma-carboxy prothrombin (DCP) [20]. Glycosylation, phosphorylation, acetylation, and ubiquitination are examples of posttranslational modifications (PTMs) that might be taken into consideration when looking for new biomarkers [21].

In this study, we looked at gene expression levels for six currently proposed HCC biomarkers, including AFP and glypican 3 (GPC3), Midkine (MDK), a disintegrin and metalloproteinase 8 (ADAM8), hepatic growth factor (HGF), and cytokeratin-1 (CK-1) in individuals with hepatocellular carcinoma, compared to serum AFP levels, to assess whether they can be used for predictive indicators or for screening in HCC Egyptian patients.

## Methods

The present study involved 70 chronic liver disease (CLD) subjects (36 men and 34 women), distributed into 2 groups: group 1 involved 40 HCC patients; group 2 involved 30 HCV subjects but no evidence of HCC. They ranged in age from 44 to 58 years. They were chosen from people who were referred to Menoufia University, Egypt’s National Liver Institute Hospital. Thirty apparently healthy subjects were divided into 15 (50.0%) males and 15 (50.0%) females, with 44 to 55 age range. This research was performed according to the national and international ethical guidelines (good clinical practice, Declaration of Helsinki), and the procedures were agreed according to the National Liver Institute Hospital Local Ethics Committee, NLI (IRB00003413) Menoufia University. All HCC and HCV subjects were positive for serum HCV, which was validated by qualitative RT-PCR to detect RNA of HCV. Ultrasonography and computed tomography (CT) scans revealed that HCC patients had a localized lesion. All of the individuals tested negative for hepatitis B surface antigen in their blood (ELISA). Only patients who granted informed consent had their blood samples taken. All patients and controls were given a thorough medical history. Patients undergoing infection with HBV or any other hepatitis diagnosable disorder rather than HCV, antiviral treatment, previous HCC, and any concomitant tumors rather than HCC were all excluded. All individuals had peripheral blood samples taken for normal assessment, which included a complete blood count (CBC), liver functions, and commercial assays for anti-HCV titer, prothrombin time, AFP, HBc-Ab, and HBsAg. EDTA-peripheral blood was taken for qRT-PCR and promptly frozen at − 80 °C until analysis.

### Quantitative real-time-PCR (qRT-PCR) for ADAM8

PureLink RNA Mini Kit (Thermos Scientific, USA) was used to isolate total RNA from peripheral blood mononuclear cells (PBMCs) according to the protocol guidelines. The RNA content and purity were defined using a NanoDrop spectrophotometer (Thermo Fisher Scientific, MA, USA). The reverse transcription was done on the isolated RNA using the HiSenScriptRH( −) cDNA synthesis kit (iNtRon, Korea) utilizing 1-μg RNA. Then, qPCR amplification was achieved in a 10-μl mixture having 1 × Universal SYBR Green RealMOD Real-Time PCR Master Mix (iNtRon, Korea), 25 ng of cDNA, and 2 pmol of each specific primer pair (Table 1) in StepOne™ Real-Time PCR Detection System (Applied Biosystem, CA, USA). Thermocycling was set to 95 °C for 10 min and then 40 cycles of 15 s at 95 °C and 1 min at 60 °C. The relative mRNA levels of the examined genes were determined using qRT-PCR, with GAPDH as an internal reference and the different biomarkers mRNA expressed as fold change. The differential expression level of various genes was normalized to the housekeeping marker (GAPDH); the cycle threshold (CT) was determined using the relative CT technique and expressed as 2^−ΔΔCT^ [22].

**Table 1:** Demographic data and biochemical and hematological characteristics of the study

Variables	The studied groups (mean ± SD)			
	**Control (** ***n*** ** = 30)**	**HCV (** ***n*** ** = 30)**	**HCC (** ***n*** ** = 40)**	***p*** **-value**
**Demographic data**				
Age: range (mean)	45.48 ± 14.2	52.4 ± 15.6	58.2 ± 10.63	NS
Gender				
Male	20 (66.7%)	17 (56.7%)	28 (70%)	NS
Female	10 (33.3%)	13 (43.3%)	12 (30%)	
**Biochemical parameters**				
ALT	18.52 ± 4.8^1^	51.06 ± 21.09^2^	85.90 ± 67.94^2^	0.000**
AST	18.84 ± 6.1^1^	41.73 ± 19.0^2^	73.15 ± 58.22^3^	0.000**
Total bil	0.45 ± 0.142^1^	0.65 ± 0.173^1^	2.18 ± 3.633^2^	0.018**
Albumin	4.478 ± 0.348^1^	4.166 ± 0.358^1^	3.36 ± 0.563^2^	0.000**
Direct bil	0.232 ± 0.090^1^	0.360 ± 0.110^1^	1.150 ± 2.124^2^	0.026*
Indir. bil	0.232 ± 0.090^1^	0.313 ± 0.097^1^	0.915 ± 1.497^2^	0.014
**Hematological parameters**				
Plat	294.5 ± 83.38^1^	194.9 ± 58.1^2^	154.0 ± 79.041^3^	0.000**
INR	1.00 ± 0.00^1^	1.05 ± 0.0770^1^	1.23 ± 0.200^2^	0.000*
WBCs	6.90 ± 1.94^1^	6.2 ± 2.34^1^	5.6 ± 2.3^1^	0.199
Hgb	14.14 ± 1.71^1^	13.7 ± 1.51^1^	12.5 ± 1.544^2^	0.005*

Different numbers bearing statistically significant

^*^means statistically significance

^**^means highly statistically significance

### Statistical analysis

The Statistical Package for the Social Sciences was utilized to examine the data (SPSS version 20.0) (Armonk, NY, USA: IBM Corp). The following assessments were utilized to test significance of variations in qualitative data (percentage and number) and quantitative statistics (standard deviation and mean). The chi-square was applied to compare differences in qualitative differences and percentages among groups. ANOVA was managed to test the variations between quantitative parametric multiple groups while nonparametric tested by Kruskal–Wallis tests. The cut-off value was determined by ROC curve. Spearman’s correlation identifies nonparametric correlation. The *P*-value cutoff for significance was determined at < 0.05.

## Results

### Demographic, hematological, biochemical, and characteristics data of the studied groups

Table 1 summarizes the hematological, demographic, and biochemical data of the groups under investigation. Healthy controls, HCV patients, and HCC patients had median ages of 45.48, 51.4, and 58.2, respectively. The HCV, HCC, and control groups did not differ significantly in terms of gender. HCC patients had considerably greater ALT levels, SGOT, AST, total bilirubin, and INR than the other studied groups, but albumin, hemoglobin, and platelet number were lower with significant value. There were no statistical significant differences among the subjects under study for creatinine or WBCs.

### Expression of the biomarkers and AFP level in the studied group

**Fig. 1:**
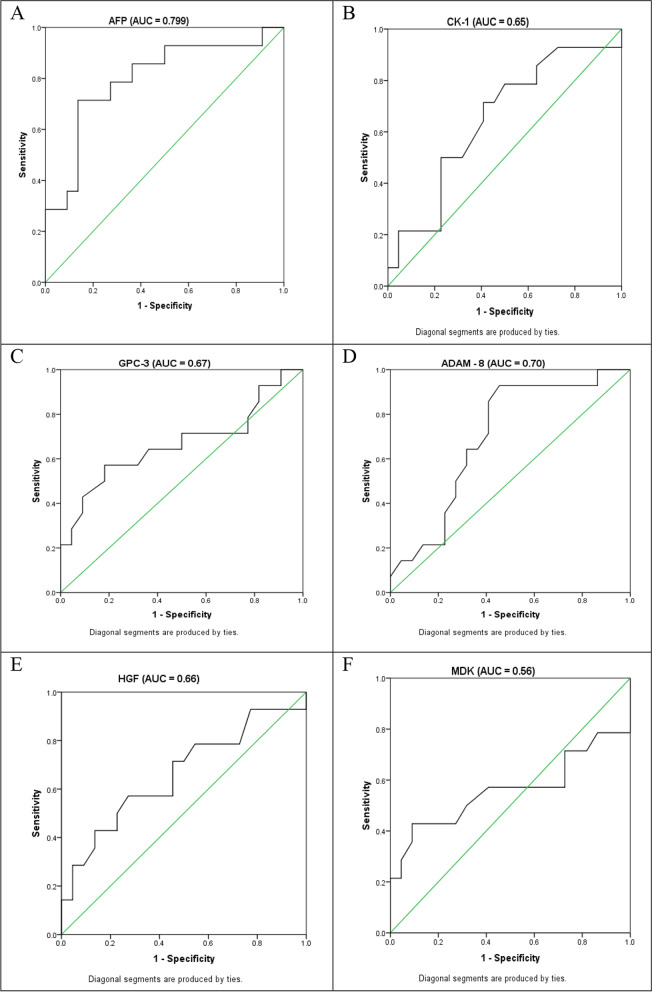
The ROC curve analysis of AFP and different biomarkers as markers for hepatocellular carcinoma. ROC, receiver operating characteristic; AFP, alpha-fetoprotein

Table 2 shows the ranges of gene expression levels identified by qRT-PCR in studied groups, standardized to the housekeeping GAPDH gene, and compared to the level of AFP in serum. The median serum AFP level demonstrated a significant rise in HCC subjects versus HCV and normal control groups (*P* = 0.001). The expression level of CK1, ADAM8, GPC3, HGF, and MDK was found significantly higher in HCV and HCC groups compared to a group of healthy controls (Fig. 1).

**Table 2:** Parameters among the studied groups

Variables Fold change	The studied groups				
	HCC *N* = 40	HCV *N* = 30	Control *N* = 30	*U*-test	*p*-value
AFP					
Mean ± SD	290.2 ± 202.7	3.38 ± 1.36	1.12 ± 0.44	3.81	0.001^1^
Median	42.5	3	1	4.11	0.001^2^
Range	3.66–1306	2–5	0.5–1.9	3.97	0.02^3^
CK-1					
Mean ± SD	1.61 ± 1.82	1.44 ± 1.36	0.44 ± 0.27	0.08	0.74^1^
Median	1.1	1.11	0.34	2.76	0.006^2^
Range	0.07–6.86	0.09–5.2	0.11–1	2.58	0.01^3^
GPC-3					
Mean ± SD	2.09 ± 1.44	1.65 ± 0.79	0.60 ± 0.25	0.52	0.61^1^
Median	2.29	1.62	0.62	2.43	0.02^2^
Range	0.35–4.92	0.41–3.03	0.29–1.15	3.24	0.001^3^
ADAM-8					
Mean ± SD	1.04 ± 0.61	0.79 ± 0.54	0.57 ± 0.51	.06	0.29^1^
Median	0.87	0.60	0.42	2.37	0.02^2^
Range	0.27–2.64	1.0–1.61	0.08–1.87	0.73	0.47^3^
HGF					
Mean ± SD	6.91 ± 6.42	4.13 ± 4.48	2.45 ± 2.49	0.93	0.35^1^
Median	4.30	2.83	1.62	1.85	0.07^2^
Range	0.11–18.38	0.13–16	0.27–8	0.99	0.32^3^
MDK					
Mean ± SD	3.78 ± 4.69	2.47 ± 1.42	1.13 ± 0.37	0.10	0.92^1^
Median	1.93	2.07	1.31	1.20	0.23^2^
Range	0.22–17.18	0.57–6.06	0.57–1.62	2.87	0.004^3^

^1^ comparison of HCC group with HCV group

^2^ comparison of HCC group with control group

^3^ comparison of HCV group with control group

### Combination of AFP and different biomarkers for potential diagnosis of HCC

**Fig. 2:**
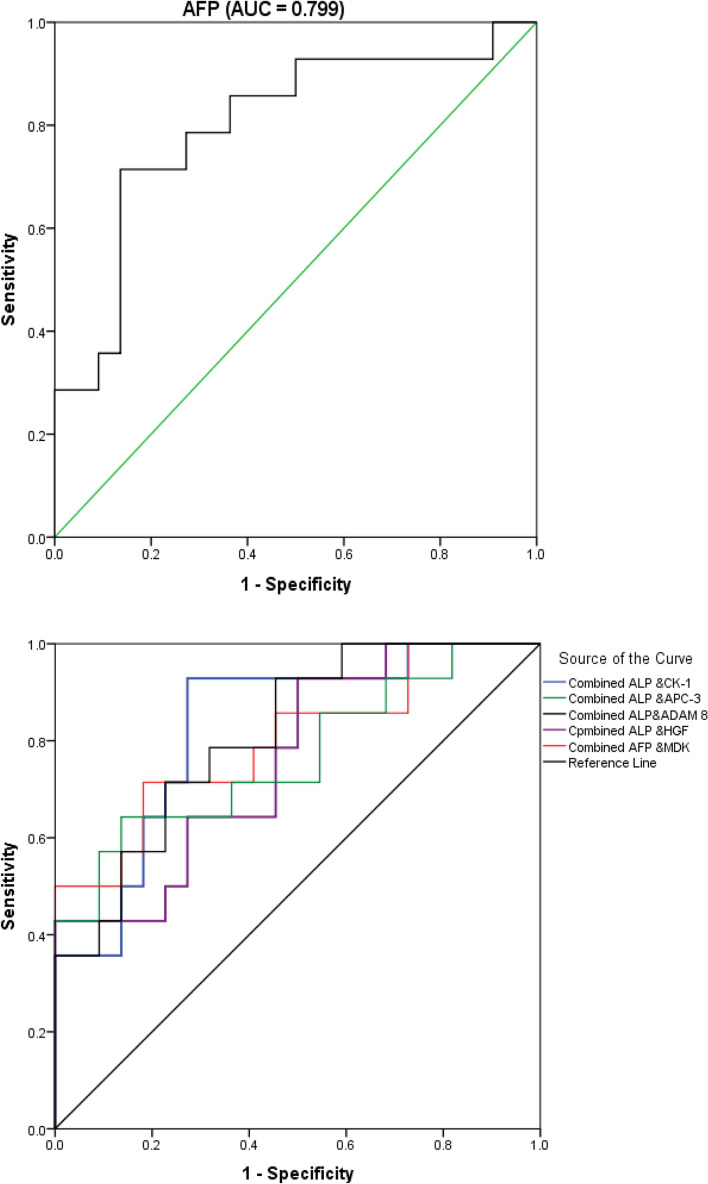
The ROC curve analysis of AFP combination with different biomarkers as markers for hepatocellular carcinoma. ROC, receiver operating characteristic

The accuracy of the noninvasive HCC biomarkers together with AFP serum expression was compared using ROC curve analysis. Table 3 shows the diagnostic ability and appropriate values of cutoff for the examined genes expression and AFP for HCC detection. ROC curve evaluation demonstrated that the AFP area under the curve (AUC), as well as its sensitivity and specificity, were higher than those of the other genes (Fig. 2), with the exception of ADAM-8, which could detect HCC at a level of 0.57-fold with 85.7% sensitivity and 60.3% specificity, whereas AFP could detect HCC at a level of 90 ng/dl with 78.7% sensitivity and 60.3%. specificity. When compared to the standard tumor marker AFP, these findings show that only ADAM8 is more sensitive but less specific in predicting HCC, implying that it will have better consequences on HCC prediction compared to HCV and the healthy control group.

**Table 3:** The diagnostic validity and the optimal cut-off values of different gene expressions and AFP as markers for HCC

Variable	Cutoff	Sens. %	Spec.%	AUC	SE	95% (*CI*)	*p-value*
AFP	7.55	78.6%	72.7%	0.799	0.08	0.64–0.95	**0.003**
CK-1	0.872	71.4%	59.1%	0.65	0.10	0.46–0.84	0.14
GPC-3	1.23	64.3	63.4%	0.67	0.10	0.47–0.87	0.10
ADAM-8	0.575	85.7%	59.1%	0.70	0.09	0.52–0.87	**0.05**
HGF	2.30	71.4%	54.5%	0.66	0.10	0.45–0.87	0.11
MDK	1.93	50%	68.2%	0.56	0.11	0.34–0.78	0.55

### Combination of the different genes with the AFP as a potential tumor marker for HCC patients

The combined application of the different genes under this study and AFP-based biomarkers has been tested to address the problem of markers being suboptimum due to poor sensitivity and specificity (Tables 4, 5). The combination of AFP and CK-1 markers used in study achieved 92.9% sensitivity and 72.8% specificity. No other combination for HCC detection showed improved sensitivity more than AFP sensitivity alone. On the other hand, when we combine the different markers without AFP, the result showed no increase of sensitivity or specificity than using AFP alone. The three combinations of AFP with two markers did not mark increase of the diagnostic ability for HCC as it raises the sensitivity in most combination from 78.6% for AFP alone to nearly 86% sensitivity, while the specificity did not improve (Fig. 2).

**Table 4:** The diagnostic validity and the optimal cut-off values of AFP and HCC marker combination

Variable	Cutoff	Sens. %	Spec.%	AUC	SE	95% (*CI)*	*p-value*
AFP	7.55	78.6%	72.7%	0.799	0.08	0.64–0.95	**0.003**
AFP & CK-1	–	**92.9%**	72.7%	0.83	0.07	0.69–0.97	**0.001**
AFP & GPC-3	–	71.4%	63.6%	0.77	0.09	0.60–0.94	**0.008**
AFP & ADAM-8	–	71.4%	77.3%	0.81	0.07	0.67–0.95	**0.002**
AFP & HGF	–	64.3%	63.6%	0.76	0.08	0.60–0.92	**0.009**
AFP & MDK	–	71.4%	81.8%	0.80	0.08	0.64–0.96	**0.003**

**Table 5:** The diagnostic validity and the optimal cut-off values of different HCC markers combination

Variables	Sensitivity	Specificity	AUC	SE	95% *CI*	*p*-value
AFP, CK-1, & GPC-3	86.4%	71.4%	0.85	0.07	0.72–0.97	**0.001**
AFP, CK-1, & ADAM8	77.3%	64.3%	0.83	0.07	0.69–0.96	**0.001**
AFP, CK-1, & HGF	77.3%	64.3%	0.80	0.07	0.66–0.95	**0.003**
AFP, CK-1, & DMK	81.8%	64.3%	0.83	0.07	0.69–0.97	**0.001**
AFP, GPC-3, & ADAM8	86.4%	64.3%	0.79	0.08	0.63–0.96	**0.003**
AFP, GPC3, & HGF	86.4%	71.4%	0.82	0.09	0.65–0.98	**0.002**
AFP, GPC3, & MDK	81.8%	78.6%	0.83	0.08	0.68–0.99	**0.001**
AFP, ADAM8, & HGF	81.8%	78.6%	0.81	0.08	0.65–0.96	**0.002**
AFP, ADAM8, & MDK	77.3%	71.4%	0.83	0.07	0.69–0.96	**0.001**
AFP, HGF, & MDK	81.8%	71.4%	0.79	0.09	0.62–0.95	**0.004**

## Discussion

HCC is a frequent and ambitious malignancy with a poor prognosis all over the world. Approximately 70 to 90% of individuals with HCC had history of cirrhosis or another chronic liver illness carried on by infection with + HBV or HCV [23]. HCV is the most common underlying etiology of liver cirrhosis, accounting for 91.32% of HCC cases, according to a major epidemiological study conducted on Egyptian HCC patients, while chronic infection of HVB was found only in 2.51% of HCC cases [24]. HCC patients with early identification and resection may have a better chance of long-term survival. Unfortunately, just 10 to 20% of patients with HCC are candidates for resection using current diagnostic methods [25]. In clinical practice, AFP is currently the only serological biomolecule for HCC. Because of its low sensitivity and nonspecific rise in noncancerous hepatic disorders, other diagnostic biomarkers are urgently needed to aid HCC early identification, particularly in AFP-normal cases and tumors of smaller sizes.

HCC pathogenesis is a multistage and multistep process that involves both genetic and environmental elements. The establishment of diagnostic biomarkers with predictive value can be aided by molecular identification of genetic abnormalities in the number and cellular content of tumor cells, as well as the microenvironment of tumor cell [26]. Multiple studies have indicated that biomarkers including MDK, ADAM-8, HGF, GPC-3, and CK1 can influence transcription via regulating contacts and signalling events in cell membrane focal adhesions, which appear to be crucial to transcriptional control [27, 28].

In many forms of solid tumors, including HCC, MDK plays an important role in tumorigenesis-related processes such as anti-apoptosis, migration, mitogenesis, angiogenesis, proliferation, and transformation [29, 30]. In this study, the diagnosis rate of both MDK and AFP was considerably greater in HCC subjects rather than subjects with healthy control individuals and HCV subjects (*P* < 0.004), but the diagnostic ability of MDK alone is still lower than AFP detection (78.7%, 72.8 vs 50%, 68%) sensitivity and specificity respectively. These data were in line with those of Shaheen et al. [31] who stated that the median MDK expression level was considerably higher in HCC patients when compared to cirrhotic subjects as well as unaffected controls.

ADAM-8 has been found to involved in multiple cellular processes. It has been linked to allergies, carcinogenesis, abnormal neural cell signalling, and arthritis, and it has been shown to be upregulated in a variety of malignancies [32, 33]. In the present investigation, the mRNA expression level of ADAM-8 and AFP levels was substantially greater in HCC patients when compared to HCV subjects and healthy individuals (*P* < 0.02), but the diagnostic validity of ADAM-8 alone is more sensitive and less specific than AFP (78.7%, 72.8 vs 85.7%, 59.1%) sensitivity and specificity respectively. These findings are consistent with Fitzmorris and Singal’s [34] study, which found that ADAM8 expression was linked to serum AFP increase, tumor size, histological differentiation, tumor recurrence, tumor metastasis, and tumor stage.

Hepatocyte growth factor (HGF) is a pleiotropic cytokine that has been linked to the pathophysiology of many malignancies by boosting cancer cell motility and invasiveness in vivo and in vitro [28]. In the current study, HGF expression levels were noticeably greater in HCC patients when compared to HCV subjects and healthy individuals (*P* < 0.02), but unfortunately, the diagnostic validity of HGF alone is less sensitive and less specific than AFP (78.7%, 72.8 vs 71.4%, 54.5%) sensitivity and specificity respectively. This comes to agree with the study done by Dong et al. [35] who stated that HGF mRNA expression levels in HCC cases increased significantly as compared to both control individuals and the CH groups.

Glypican-3 (GPC3) is thought to have a vital controlling function in cell growth in mesodermal embryonic tissues. In mechanism way, GPC3 is possible to be implicated in the regulation of Wnt, bone morphogenic protein, and signalling pathways because it influences growth and death of the cell in particular types throughout development [36, 37]. In the current study, compared to patients with HCV, healthy individuals, and patients with HCC, patients with HCC had considerably greater levels of GPC-3 expression (*P* < 0.001), but unfortunately, the diagnostic validity of HGF alone is less sensitive and less specific than AFP (78.7%, 72.8 vs 64.3, 63.4%) sensitivity and specificity respectively. In the same way, GPC3 expression has been found in a variety of malignancies, including lung squamous cell carcinoma (SqCC), ovarian carcinoma, gastric carcinoma, and melanomas, and it is especially prominent in HCC [38].

Cytokeratin mRNA expression is likely to be modified in correlation with surges in malignancy and metastatic ability and a number of cytokeratins thought to be utilized as prognostic indicators in several epithelial cancers. Several cytokeratin subtypes have been found to be expressed in HCC [39]. In this study, the AFP (gold standard marker) sensitivity and specificity were 39% and 100% at 90 IU/ml cutoff, while these values are still higher than the sensitivity and specificity of CK-1 detection of HCC (71.4%, 59.1%). Bessa et al. [40] had comparable data (*AUC* 0.71) to the current results (*AUC* 0.79) for identifying HCC by measurement of AFP in Egyptian subjects with HCV-dependent HCC. Therefore, it seems that there was no ideal biomarker which has sensitivity and specificity more than the gold standard AFP in detection of HCC; in such case, there is a requirement to improve the HCC detection utilizing AFP. Our research looked into the idea of combining numerous markers to aid in the HCC detection by utilizing AFP. The CK-1 combination with AFP increased the sensitivity of HCC detection from 71.4% for CK-1 and 78.6% for AFP to 92%, while no improvement of specificity was achieved. The three combinations of AFP with two markers did not mark the increase of the diagnostic ability for HCC as it raises the sensitivity in most combination from 78.6% for AFP alone to nearly 86% sensitivity, while the specificity did not improve.

## Conclusions

In conclusion, the use of different biomarkers overlaying such as CK1 with AFP could improve the HCC detection. The ability to identify HCC patients from unaffected normal individuals and liver cirrhosis subjects, a category at high risk, provides hope for early identification of HCC cases. In order to confirm that those markers can be used, more research is needed to verify its efficiency for various populations.

## Data Availability

The datasets used during the present study are available from the corresponding author upon request.

## Abbreviations

HCC: Hepatocellular carcinoma
HCV: Hepatitis C virus
HBV: Hepatitis B virus
AFP: Alpha-fetoprotein
GPC3: Glypican-3
MDK: Midkine
ADAM8: A disintegrin and metalloproteinase 8
HGF: Hepatic growth factor
CK-1: Cytokeratin-1
DEGs: Differentially expressed genes
PBMCs: Peripheral blood mononuclear cells
qRT-PCR: Quantitative real-time PCR
CT: The cycle threshold
GP73: Golgi protein-73
Hsp90: Heat shock protein 90
AFU: Alpha-1-fucosidase
DKK1: Dikkopf-1
DCP: Des-gamma-carboxy prothrombin
